# Identification of pathways associated with chemosensitivity through network embedding

**DOI:** 10.1371/journal.pcbi.1006864

**Published:** 2019-03-20

**Authors:** Sheng Wang, Edward Huang, Junmei Cairns, Jian Peng, Liewei Wang, Saurabh Sinha

**Affiliations:** Department of Computer Science, University of Illinois at Urbana-Champaign, Urbana, Illinois, United States of America; University of Chicago, UNITED STATES

## Abstract

Basal gene expression levels have been shown to be predictive of cellular response to cytotoxic treatments. However, such analyses do not fully reveal complex genotype- phenotype relationships, which are partly encoded in highly interconnected molecular networks. Biological pathways provide a complementary way of understanding drug response variation among individuals. In this study, we integrate chemosensitivity data from a large-scale pharmacogenomics study with basal gene expression data from the CCLE project and prior knowledge of molecular networks to identify specific pathways mediating chemical response. We first develop a computational method called PACER, which ranks pathways for enrichment in a given set of genes using a novel network embedding method. It examines a molecular network that encodes known gene-gene as well as gene-pathway relationships, and determines a vector representation of each gene and pathway in the same low-dimensional vector space. The relevance of a pathway to the given gene set is then captured by the similarity between the pathway vector and gene vectors. To apply this approach to chemosensitivity data, we identify genes whose basal expression levels in a panel of cell lines are correlated with cytotoxic response to a compound, and then rank pathways for relevance to these response-correlated genes using PACER. Extensive evaluation of this approach on benchmarks constructed from databases of compound target genes and large collections of drug response signatures demonstrates its advantages in identifying compound-pathway associations compared to existing statistical methods of pathway enrichment analysis. The associations identified by PACER can serve as testable hypotheses on chemosensitivity pathways and help further study the mechanisms of action of specific cytotoxic drugs. More broadly, PACER represents a novel technique of identifying enriched properties of any gene set of interest while also taking into account networks of known gene-gene relationships and interactions.

## Author summary

Gene expression levels have been used to study the cellular response to drug treatments. However, analysis of gene expression without considering gene interactions cannot fully reveal complex genotype-phenotype relationships. Biological pathways reveal the interactions among genes, thus providing a complementary way of understanding the drug response variation among individuals. In this paper, we aim to identify pathways that mediate the chemical response of each drug. We used the recently generated CTRP pharmacogenomics data and CCLE basal expression data to identify these pathways. We showed that using the prior knowledge encoded in molecular networks substantially improves pathway identification. In particular, we integrate genes and pathways into a large heterogeneous network in which links are protein-protein interactions and gene-pathway affiliations. We then project this heterogeneous network onto a low-dimensional space, which enables more precise similarity measurements between pathways and drug-response-correlated genes. Extensive experiments on two benchmarks show that our method substantially improved the pathway identification performance by using the molecular networks. More importantly, our method represents a novel technique of identifying enriched properties of any gene set of interest while also taking into account networks of known gene-gene relationships and interactions.

This is a *PLoS Computational Biology* Methods paper.

## Introduction

Large-scale cancer genomics projects, such as the Cancer Genome Atlas [1], the Cancer Genome project [2], and the Cancer Cell Line Encyclopedia project [3], and cancer pharmacology projects, such as the Genomics of Drug Sensitivity in Cancer project [2], have generated a large volume of genomics and pharmacological profiling data. As a result, there is an unprecedented opportunity to link pharmacological and genomic data to identify therapeutic biomarkers [4–6]. In pursuit of this vision, significant efforts have been invested in identifying the genetic basis of drug response variation among individual patients. For instance, a recent study performed a comprehensive survey of genes with basal expression levels in cancer cell lines that correlate with drug sensitivity, revealing potential gene candidates for explaining mechanisms of action of various drugs [7].

While significant efforts have focused on specific genes that interact with compounds and confer observed cellular phenotypes, there has been relatively little progress in studying the synergistic effects of genes. These effects are key factors in comprehensively deciphering the mechanisms of action of compounds and understanding complex phenotypes [8]. Similarly, pathways, which comprise a set of interacting genes, have emerged as a useful construct for gaining insights into cellular response to compounds. Analysis at the pathway level not only reduces the analytic complexity from tens of thousands of genes to just hundreds of pathways, but also contains more explanatory power than a simple list of differentially expressed genes [9]. Consequently, an important yet unsolved problem is the effective identification of pathways mediating drug response variation. Although the associated pathways for certain drugs have been studied experimentally [10–12], *in vitro* pathway analysis is costly and inherently difficult, making it hard to scale to hundreds of compounds.

Fortunately, a growing compendium of genomic, proteomic, and pharmacologic data allows us to develop scalable computational approaches to help solve this problem. Although statistical significance tests and enrichment analyses can be naturally applied to compound-pathway association identification (e.g., by testing the overlap between pathway members and differentially expressed genes), these approaches fail to leverage well-established biological relationships among genes [13–16]. Even when analyzing individual genes, molecular networks such as protein-protein interaction networks have been shown to play crucial roles in understanding cellular drug response [8, 17–20]. Therefore, we propose to combine molecular networks with gene expression and drug response data for pathway identification. However, integrating these heterogeneous data sources is statistically challenging. Moreover, networks are high-dimensional, incomplete, and noisy. Thus, our algorithm needs to accurately and comprehensively identify pathways while exploiting suboptimal networks.

Here, we present PACER, a novel, network-assisted algorithm that identifies pathway associations for any gene set of interest. PACER first constructs a heterogeneous network that includes pathways and genes, pathway membership information, and gene-gene relationships such as protein-protein physical interaction. It then applies a novel dimensionality reduction algorithm to this heterogeneous network to obtain compact, low-dimensional vectors for pathways and genes in the network. Pathways that are topologically close to the given set of genes (e.g., drug response-related genes) in the network are co-localized with those genes in this low-dimensional vector space. Hence, PACER ranks each pathway based on its vector’s proximity to vectors representing the given genes. We used the proposed algorithm to discover chemosensitivity-related pathways, by applying it to genes whose basal expression level correlates with drug sensitivity. We evaluated PACER’s ability to identify compound-pathway associations with two “ground truth” sets built from compound target data [7] and LINCS differential expression data [21]. When comparing PACER to state-of-the-art methods that ignore prior knowledge of interactions among genes, we observed substantial improvement of the concordance with the chosen benchmarks. Even though we developed PACER and tested its ability to identify compound-pathway associations, the algorithm is applicable to any scenario in which one seeks to discover pathways related to a pre-specified gene set of interest, while utilizing a given gene network.

## Methods

### Compound response data and gene expression data

We obtained a large-scale compound response screening dataset from Rees *et al*. [7], which spans 481 chemical compounds and 842 human cancer cell lines encompassing 25 lineages. These 481 compounds were collected from different sources including clinical candidates, FDA-approved drugs and previous chemosensitivity profiling experiments. Area under the drug response curve (AUC) was used by the authors of that study to measure cellular response to individual compounds. We also obtained gene expression profiles for these cell lines from the Cancer Cell Line Encyclopedia (CCLE) project [22], profiled using the GeneChip Human Genome U133 Plus 2.0 Array. Since these expression measurements were done in each cell line without any drug treatment, they are referred to as “basal” expression levels. In contrast, the expression profiling of a cell line was performed after treatment with a drug in certain studies such as LINCS L1000 [21] and CMAP [23]. We obtained the SMILE specification of each drug from PubChem [24].

### STRING-based molecular network and NCI pathway collection

We obtained a collection of six human molecular networks from the STRING database v9.1 [25]. These six networks include experimentally derived protein-protein interactions, manually curated protein-protein interactions, protein-protein interactions transferred from model organism based on orthology, and interactions computed from genomic features such as fusion-fusion events, functional similarity, and co-expression data. There are 16,662 genes in the network. We used all of the STRING channels except “text-mining” and used the Bayesian integration method provided by STRING. Since our approach can deal with different edge weights, we did not set a threshold to remove low-confidence edges. We referred to this integrated network as the “STRING-based molecular network”.

To test whether genes that are highly correlated with many compounds tend to have higher degrees in the network, we formed two groups of genes. One group contained genes that are correlated with over 100 compounds, and the other group contained the remaining genes. We then used the Wilcoxon signed-rank test to test whether the degrees of genes in these two groups were from the same distribution.

We obtained a collection of 223 cancer-related pathways from the National Cancer Institute (NCI) pathway database [26]. These manually curated pathways include human signaling and regulatory pathways as well as key cellular processes.

### The PACER framework

PACER integrates pathway information with the STRING-based molecular network described above by constructing a heterogeneous network of genes and pathways. An edge exists between two genes if they are connected in the network. An edge exists between a pathway and a gene if the gene belongs to the pathway. There are no direct pathway-pathway edges in the heterogeneous network.

Formally, let *A* denote the weighted adjacency matrix of the STRING-based molecular network with n genes (or proteins). Let *B* ∈ {0, 1}^*n*×*m*^ denote the gene pathway association matrix, where *B*_*ij*_ = 1 if gene *i* is in pathway *j*. The heterogeneous network $H\in R^{\left(n+m)\times (n+m\right)}$ is then defined as:

$$\begin{array}{c} H_{ij}=\{\begin{array}{cc} A_{ij}, & i\leq n,j\leq n\\ B_{i-n,j}^{T}, & i>n,j\leq n\\ B_{i,j-n}, & i\leq n,j>n\\ 0, & i>n,j>n \end{array} \end{array}$$ (1)

PACER adopts diffusion component analysis (DCA), a recently developed network representation algorithm to learn a low-dimensional vector for each node in the network [27]. Because of its ability to handle noisy and missing edges in the biological network, DCA has achieved state-of-the-art results in several computational biology tasks [27, 28]. DCA takes *H* as input. It outputs the *d*-dimensional vectors $V\in R^{(n+m)\times d}$ for each node in *H*. According to the definition of *H*, the first *n* columns of *H* are the embedding vectors for genes. The remaining columns of *H* are the embedding vectors for pathways.

Since compounds are not nodes in the constructed heterogeneous network, only genes and pathways are projected onto the low-dimensional space. After learning the low-dimensional representations of all nodes (genes and pathways), PACER ranks pathways based on the cosine similarities between the low-dimensional representations of the pathway and a set of genes most correlated with response to a compound. Formally, the PACER score *s*_*ij*_ between pathway *i* and compound *j* is defined as:

$$\begin{array}{c} s_{ij}=\sum_{k\in \text{RCG}(j)}w_{k}\cdot \text{cos}\left(V_{k},V_{i+n}\right), \end{array}$$ (2)

Here, *w*_*k*_ is the weight for gene *k*. PACER can take input gene weights to weight these cosine similarities. In this paper, we weight the cosine similarities by using the Pearson correlation between the gene expression vector and the chemosensitivity vector. We further calculate an empirical *p*-value for each compound-pathway association. For a given drug with *n* response-correlated genes, we use a new, randomly generated set of *n* genes and compute its pathway association scores using PACER. This is repeated *k* = 10, 000 times. With *m* pathways, we then have a total of *km* PACER scores. The empirical *p*-value of each original drug-pathway PACER score is its (fractional) rank in this set of PACER scores from random gene sets.

### LINCS drug perturbation profiles

LINCS is a data repository of over 1.3 million genome-wide expression profiles of human cell lines subjected to a variety of perturbation conditions, which include treatments with more than 20 thousand unique compounds at various concentrations. Each perturbation experiment is represented by a list of differentially expressed genes that are ranked based on *z*-scores of perturbation expression relative to basal expression. For each gene, we first took the difference between its expression in a perturbation condition and its expression in a control condition (i.e., treatment with pure DMSO solvent). We then considered the differential expression of the gene in multiple perturbation experiments involving that compound (i.e., different concentrations, time points, and cell lines). We used the maximum differential expression to represent the compound’s effect on that gene’s expression. All genes were then ranked by their differential expression on treatment with the compound, and the top 250 genes were treated as differentially expressed genes (DEGs) of the compound, provided their *z*-score has an absolute value greater than 2.

### Comparison with method of Huang *et al*.

We implemented the method of Huang *et al*. [13] ourselves using the exact same input (i.e., chemosensitivity and gene expression data) as PACER. We first computed a gene’s correlation to a drug by calculating the Pearson correlation coefficient between the gene’s expression values and the drug response values across cell lines. Let the set of genes in pathway *p* be denoted by *G*_*p*_, and their correlation values to a drug *d* by *C*(*G*_*p*_, *d*). Conversely, the set of genes not in pathway *p* is denoted as $\bar{G_{p}}$, and their correlation values to *d* as $C(\bar{G_{p}},d)$. We then performed the Kruskal-Wallis *H* test, following Huang *et al*., to test if the medians of *C*(*G*_*p*_, *d*) and $C(\bar{G_{p}},d)$ were significantly different. We used the resulting *p*-value to rank pathways for each drug.

## Results

### Global analysis of correlations between basal gene expression and compound response

**Fig 1:**
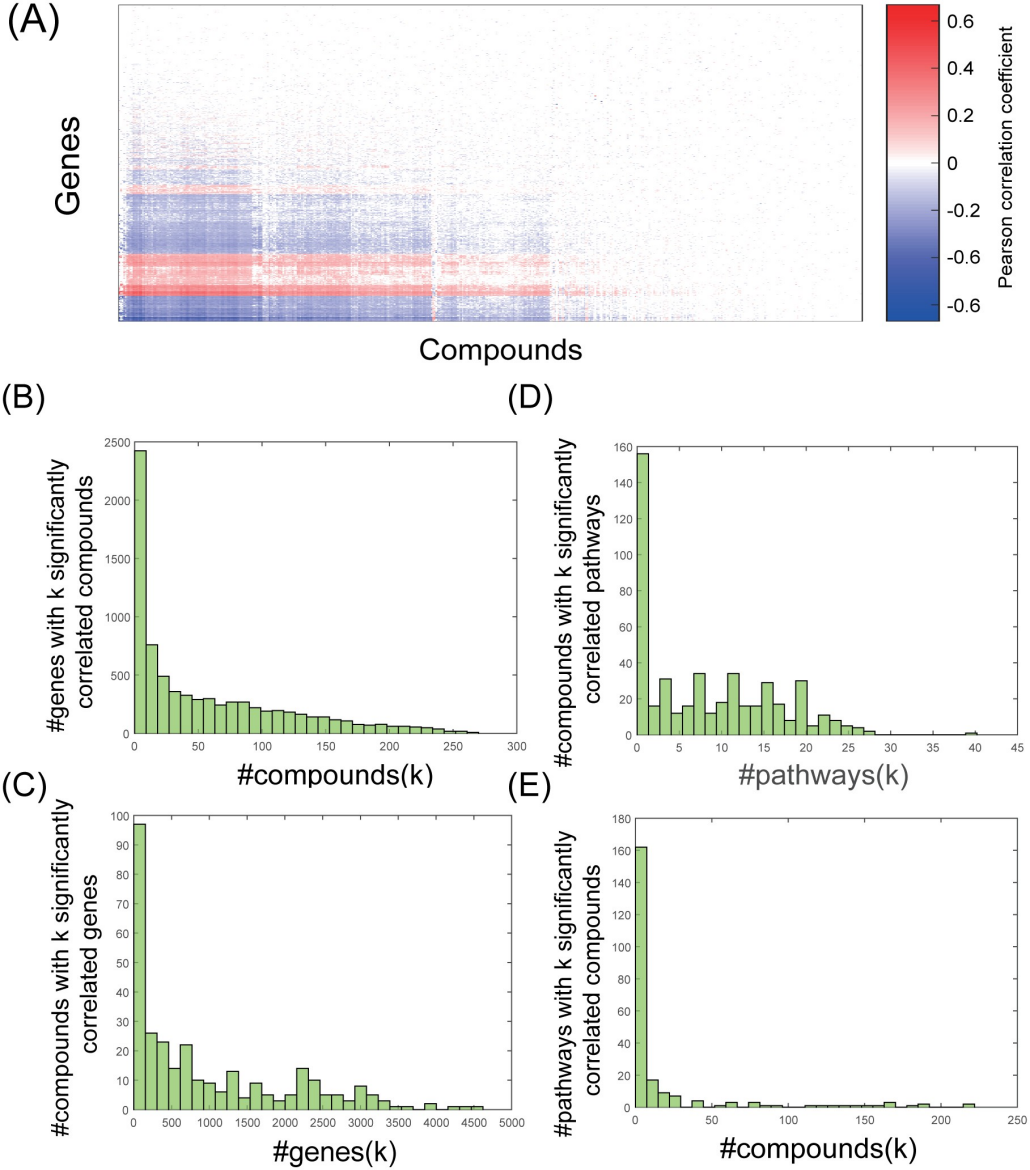
Global analysis of correlations between basal gene expression and compound response. (A) Heatmap of the Pearson correlation coefficient between genes (expression) and compounds (chemosensitivity, measured by AUC values). (B) Histogram of the number of compounds associated with each gene. The *y*-axis shows the number of genes associated with *k* compounds, where *k* is shown on the *x*-axis. (C) Histogram of the number of genes associated with each compound. The *y*-axis shows the number of compounds associated with *k* genes, where *k* is shown on the *x*-axis. (D) Histogram of the number of compounds significantly associated with each pathway (Fisher’s exact test FDR < 0.05). (E) Histogram of the number of pathways significantly associated with each compound (Fisher’s exact test FDR < 0.05).

Following the work of Rees *et al*. [7], we first examined correlations between the compound sensitivity and basal gene expression profiles across hundreds of cell lines. We calculated Pearson correlation coefficients between each gene’s expression and the cellular response to each compound (measured as AUC, see Methods), across different cell lines (Fig 1A). In contrast to IC50 and EC50 scores, AUC simultaneously captures the efficacy and potency of a drug. Of the 8.7 million pairs of genes and compounds tested, we found 294,789 to be significantly correlated (*p*-value < 0.0001 after Bonferroni correction, corresponding to a Pearson correlation coefficient of 0.215.) Since the Rees *et al*. dataset comprises measurements on 842 cell lines, each correlation was computed over 842 pairs of values (drug response, gene expression pairs). This is why even a modest-looking Pearson correlation of 0.215 was deemed highly statistically significant. The key observation from this initial analysis, also noted by Rees *et al*., is that basal gene expression levels are highly correlated with cytotoxic response for large numbers of compound-gene pairs. Within these significantly correlated pairs, 26 genes were correlated with over 250 compounds (Fig 1B, S1 Table). We note that these key genes tend to be high-degree nodes in the STRING-based molecular network (Wilcoxon rank-sum test *p*-value < 9.6e-14, see Methods). We also found that some (10 of 481) compounds were significantly correlated (Pearson correlation *p*-value < 0.0001 after Bonferroni correction) with more than 3,200 genes (Fig 1C). Five of these ten compounds are chemotherapeutic agents (S2 Table). In contrast, about 100 compounds were not significantly correlated with any genes; these compounds are mostly probes that either lack FDA approval or are not clinically used. The large disparity among the examined compounds in terms of the number of correlated genes reflects the diversity of these 481 small molecules. While many of them are chemotherapeutic, which can affect the expression of a large number of genes, some compounds may be targeting specific mutations, post-translational modifications, or protein expression. A closer examination revealed that the compounds with the highest AUC had the fewest gene correlations (i.e., fewest genes whose expression correlates with cytotoxic response) (Fig 1 in S1 Text). This suggests that the strategy of identifying compound-associated genes by correlating basal gene expression profiles with cytotoxicity is likely to be more effective for more potent compounds, for which average response is stronger. Note that the gene expression profiles used here are basal and not in response to treatment with compound, hence it was not clear *a priori* that more effective compounds would have larger numbers of gene correlates. In summary, examination of individual genes’ correlations with chemical response confirmed previous reports [2, 7, 29] that basal gene expression is significantly correlated with cytotoxicity across cell lines, especially for effective cytotoxic drugs. For each compound, we refer to the top 250 genes whose expression are most significantly correlated with chemosensitivity (Pearson correlation *p*-value < 0.0001 after Bonferroni correction) as “response-correlated genes” (RCGs) for this compound.

### Identifying compound-specific pathways via enrichment tests

**Fig 2:**
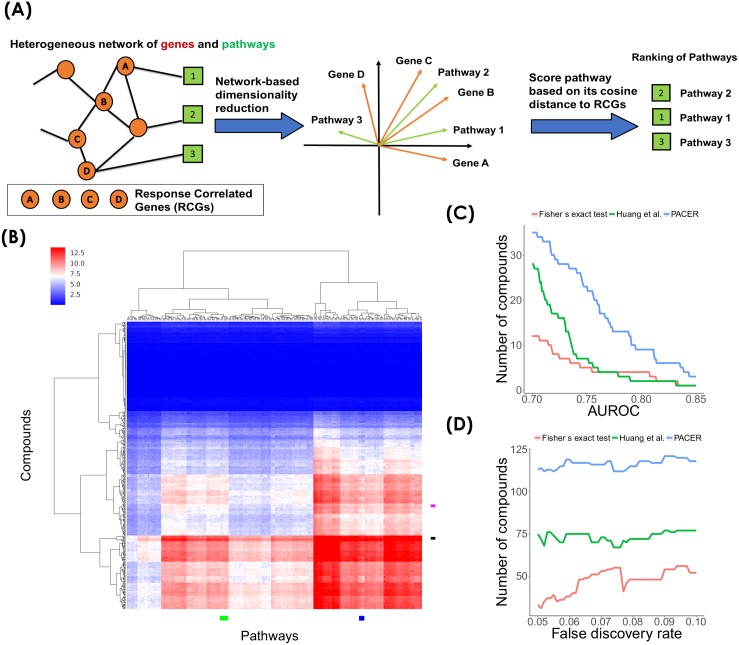
Identifying pathways associated with chemical response using PACER. (A) Schematic description of PACER. (B) Heatmap of associations between compounds and pathways (PACER scores). Columns are compounds and rows are pathways. (C) Comparative evaluation of different methods for predicting compound-pathway associations. The ground truth used here is the pathways that contain any known target gene of the compound. (D) Number of compounds with significant overlap (*p* < 0.05) between pathways from LINCS and pathways from PACER, from Huang *et al*. 2005 and from the baseline method (Fisher’s exact test) respectively, at different levels of stringency in pathway prediction. Stringency refers to the FDR control used by the baseline method in determining significant pathways. Both PACER and the Huang *et al*. 2005 method were used to predict the same number of (highest scoring) pathways as the baseline method, for a fair comparison.

The above evidence for correlations between basal gene expression and chemical response raised the possibility that one might discover important biological pathways associated with the response by a systems-level analysis of gene expression data. To explore this, we considered a collection of 223 cancer-related pathways from the National Cancer Institute (NCI) pathway database [26] and used Fisher’s exact test to quantify the overlap between the set of genes in a given pathway and RCGs. A significantly large overlap between the two sets indicates an association between the pathway and the compound. We performed a multiple hypothesis correction on all pathway association tests for each compound, using FDR = 0.05. The results of this baseline method for predicting pathway associations are shown in Fig 1D (distribution of the number of compounds that are significantly associated with each pathway) and Fig 1E (distribution of the number of pathways significantly associated with each compound). Both distributions revealed a long tail. For instance, while each pathway was associated with an average of 18 compounds (of the 481 tested), there were 10 pathways that were associated with over 150 compounds (S3 Table). Likewise, while each compound was associated with an average of eight pathways, there were 12 compounds associated with over 25 pathways (S4 Table). We show the details of these long tails in Fig 2 in S1 Text.

### A new method for identifying pathways associated with chemical response, based on network embedding

We observed above that key RCGs (i.e., those correlated with many compounds) tend to be enriched in high degree nodes in the STRING-based molecular network. This suggests that an analysis combining this network with pathway enrichment tests might provide additional insights. We therefore developed a novel network-based method, called PACER, for scoring compound-pathway associations. PACER (Fig 2A) first constructs a heterogeneous network consisting of genes and pathways as nodes. In this network, gene-pathway edges denote pathway memberships based on a compendium of pathways and gene-gene edges from the STRING-based molecular network introduced above (also see Methods). PACER then creates a low-dimensional vector representation for each gene and pathway node in the heterogeneous network, reflecting the node’s position in this heterogeneous network. This is done by the Diffusion Component Analysis (DCA) approach reported in previous work [27, 28]. Nodes (i.e., pathways or genes) will have similar vector representations if they are near each other in the network. For instance, two pathway nodes will have similar vector representations if the pathways share genes and/or their genes are related in the STRING-based molecular network. In a similar vein, two genes will have similar representations if they belong to the same pathway(s) and/or possess the same network neighbors. A gene and a pathway can also be compared in the low-dimensional space, and will be deemed similar if the gene is in the pathway and/or the gene is related in the network to other genes of the pathway. Using the low-dimensional vectors calculated by DCA, PACER next scores a pathway based on the average cosine similarity between the vector representation of the pathway and those of the RCGs. A pathway can thus be found to be associated with a compound if, in the network, the pathway genes are closely related to the compound’s RCGs; this association can be discovered even if the pathway does not actually include the RCGs. We note that scores assigned by PACER are not statistical significance scores and are meant only to rank pathways for association with a given compound. Also, a negative score assigned to a compound-pathway pair does not imply a negative correlation between expression levels of pathway genes and chemosensitivity. Rather, it only implies a lack of evidence for an association between the compound-pathway pair. Since pathway association analysis is likely to be meaningless for compounds with very few RCGs, we limited the following analysis to the 330 compounds for which more than 5 RCGs were identified.

The PACER association scores for all combinations of 330 compounds and 223 NCI signaling pathways are shown in Fig 2B. Since PACER scores are not easily assigned statistical significance levels, we chose to examine, for each compound, the *n* pathways with the highest PACER scores, where *n* is the number of statistically significant pathway associations (FDR < 0.05) found by the baseline method above for the same compound. (This choice also allows a fair comparison between the two methods in subsequent sections.) We found literature support for several of these associations. For example, PACER analysis associates ruxolitinib, a JAK/STAT inhibitor, with integrin-linked kinase signaling pathway. In a previous study, it was shown that beta 4 integrin enhances activation of the transcription factor STAT3, which is a target of ruxolitinib [30]. Fig 2B reveals that the pathways cluster into many distinct groups, each with different compound association profiles. In some cases, we noted functionally related pathways being grouped together. For example, one group consists of pathways describing various integrin cell surface interactions including “integrin family cell surface interactions”, “alpha E beta 7 integrin cell surface interactions”, “alpha 6 beta 4 integrin-ligand interactions”, and “beta 5 beta 6 beta 7 and beta 8 integrin cell surface interactions” (marked as blue rectangle in Fig 2B). These pathways are known to play crucial roles in communications among cells in response to small molecules [31]. Notably, the integrin-mediated pathways promote invasiveness and oncogenic survival, and contribute to cancer cell survival and resistance to chemotherapy [32, 33]. Another group consists of different interleukin signaling pathways including “IL4-mediated signaling events”, “IL8- and CXCR1-mediated signaling events”, “IL3-mediated signaling events”, and “IL2 signaling events mediated by PI3K” (marked as green rectangle in Fig 2B). Our analysis found that this group of pathways is associated with decitabine. A recent study shows that decitabine’s effect of PD-1 blockade-based immunotherapy is enhanced in colorectal cancer through upregulation of many immune-related genes [34].

Fig 2B also shows compounds clustered into different groups based on their associations with pathways. We noted examples where many compounds with similar structures were grouped together. For example, teniposide and etoposide had a Tanimoto similarity score of 0.94 between their SMILE specifications, which was substantially higher than the average Tanimoto similarity score of 0.3716 for all pairs of compounds. They were clustered together in the same group (marked as black rectangle in Fig 2B), which had seven compounds. Among the pathways that are associated with this group, we found a set of similar pathways, including “p53 pathway”, “direct p53 effectors”, “signaling mediated by p38-alpha and p38-beta”, and “signaling mediated by p38-gamma and p38-delta”. We found support in the literature in favor of some of these associations. For example, a previous study reported that etoposide activates p38MAPK and can be used as a combined treatment approach when used with p38MAPK inhibitor SB203580 [35]. As another example, temsirolimus and tacrolimus, which are both epipodophyllotoxins and inhibit topoisomerase II, have a Tanimoto similarity score of 0.82, and are grouped closely in Fig 2B (marked as pink rectangle in Fig 2B).

### PACER improves pathway identification

We noted a substantial degree of complementarity between the top predictions of PACER and those of the baseline method that uses Fisher’s exact test between RCGs and pathway genes (see S5 Table). For instance, PACER found that bexarotene is associated with the “IGF1 pathway”. A recent study showed that treating rats with high doses of bexarotene substantially decreased serum IGF1 levels [36]. The baseline approach did not find this association to be significant. Similarly, PACER reported that the “ATM pathway” is associated with simvastatin, while the baseline method did not. Simvastatin has been reported to activate ATM when it is used to treat chronic lymphocytic leukemia patients [37].

For a more systematic comparison between the two methods, we evaluated PACER based on a database of known compound targets. We performed the evaluation under the assumption that a pathway containing at least one known target is an associated pathway. Huang *et al*. suggested and used this approach [13]. We used it here to evaluate PACER, the baseline method, as well as a third method presented by Huang *et al*. [13] Although this third method was proposed to detect associations between pathways and drug clades, it can directly detect pathway-compound associations. We implemented the method ourselves (see Methods) and included it in our evaluations. We obtained the known targets for our compound set from Rees *et al*. [7] and STITCH database [38]. We then computed the AUROC of pathway predictions made by PACER for each compound, and plotted this information alongside analogous information for the baseline method and the method of Huang *et al*. [13] As shown in Fig 2C, PACER identified pathways with higher AUROC compared to the other two methods. For example, PACER identified pathways with an AUROC greater than 0.75 for 23 different compounds, while the baseline method achieved this level of AUROC for only 5 compounds. Table 1 shows the 10 compounds for which PACER achieved highest AUROC (Fig 4-7 in S1 Text).

**Table 1:** Compounds for which PACER predicted pathways with greatest AUROC. Evaluation was performed with known targets.

Compound	AUROC
bms-536924	0.868778
raf265	0.859769
pf-3758309	0.858974
nsc23766	0.843931
nilotinib	0.838382
kx2-391	0.835238
bosutinib	0.813179
mk-2206	0.811792
pf-573228	0.811445
zstk474	0.794220

We further compared the associations predicted by the three methods to those identified from an external data set. We mined the Library of Integrated Network-Based Cellular Signatures (LINCS) L1000 data [21], which reports genes differentially expressed upon treatment of various cell lines with a compound. For each compound in our analysis that is also included as a perturbagen in the L1000 compendium, we established a LINCS-based benchmark of significantly associated pathways. This was based on a Fisher’s exact test (*p*-value < 0.05) between pathway genes and the most differentially expressed genes from treatments with the same compound (see Methods). We required this criterion to be met in at least one of the cell lines for which data was available from LINCS. We then assessed the concordance between this set of LINCS-based compound-pathway associations and those predicted by either method presented above. We recognize that this is not an ideal benchmark: LINCS data points to genes (and, indirectly, to pathways) that are differentially expressed in response to treatment, while PACER and the compared methods base their pathway predictions on genes that have basal expression levels across cell lines that correlate with chemical response. At the same time, we expect the pathways affected by chemical treatment to also be, to an extent, involved in interpersonal variation of chemosensitivity, making this a suitable evaluation procedure. This was inspired by similar observations in cancer biology: genes and pathways disrupted in cancer tissues overlap with genes and pathways whose mutation status in germline non-tumor samples is informative about disease susceptibility and progression.

To test whether the significant pathways identified from LINCS data agree with the pathways predicted by one of the methods being evaluated (based on chemical response variation in CCLE cell lines), we counted the compounds for which the two sets of predicted pathways overlapped significantly (Fisher’s exact test *p*-value < 0.05). As shown in Fig 2D, the PACER approach predicts pathways concordant with the corresponding LINCS-based benchmark for more compounds, compared to the baseline method and that of Huang *et al*. [13] For instance, when the baseline method used an FDR threshold of 10% to designate significant pathway associations for each compound, and the PACER method predicted the same number of pathways, the latter’s predictions were concordant with the LINCS-based benchmark for 118, a nearly two-fold improvement over the baseline method’s predictions. Our evaluations actually provide evidence for the above-mentioned possibility that pathways predictive of drug sensitivity overlap with genes that mediate drug response. In fact, we found 113 compounds for which the pathways identified from basal expression correlations and the pathways identified from LINCS signatures overlap with FDR < 5%.

After observing the substantial improvement of PACER, we then investigated whether the performance of PACER is stable when using only experimental derived protein-protein interactions as input. We found that this is indeed the case, as per the two evaluation strategies presented above (Fig 8-9 in S1 Text). We further demonstrated that the result of our method is robust to different numbers of top response-correlated genes used in PACER, as shown in Fig 10-11 in S1 Text. We compared different values for ‘k’ in the ‘top k’ genes chosen by PACER. We found that results were comparable when using k = 100, 150, 200, 250 and 300. This demonstrates the stability of the algorithm’s performance to different but reasonable values of k in its choice of top k response-correlated genes.

## Discussion

We have shown that embedding prior knowledge in a gene network can more accurately identify compound-pathway associations. Our new method, called PACER, identified many compound-pathway associations that are supported by known compound targets as well as literature evidence. Due to its unique ability to incorporate any suitable compendium of gene interactions, our approach may provide complementary insights into drug mechanisms of action.

Historically, pathways associated with a particular gene set are identified by using popular statistical methods such as Gene Set Enrichment Analysis [39], Fisher’s exact test, or the Binomial test (Reactome [40]). These tools test the overlap between differentially expressed genes and pathway members. They may also be applied to the set of drug-response-correlated genes (RCGs) analyzed here. Ingenuity Pathway Analysis [41] is another related tool, which utilizes information about causal interactions between pathway members. Our study is similar to the above tools in that PACER also seeks to find pathways implicated by a gene set. However, our approach differs from these existing tools in that known molecular interactions (e.g., PPI) among different genes are taken into consideration. Thus, a gene set, be it the RCGs of a compound or the members of a pathway, is not treated merely as the sum of its parts, but also includes the relationships among those parts. Since the dominant theme in existing approaches is assessment of overlaps between two gene sets (MSigDB, DAVID, and Reactome adopt variations on this theme), our extensive comparisons between PACER and the baseline method of Fisher’s exact test shed light on the relative merits of the new approach. A related line of work aims to identify differentially expressed subnetworks in a given interaction network, e.g., KeyPathwayMiner [42], but these studies are only superficially relevant to our work since we aim to prioritize existing pathways instead of finding new pathways.

We consider two potential reasons for the strong performance of PACER. First, it is widely appreciated that a chemical compound not only affects individual genes, but also combinations of genes in molecular networks corresponding to core processes, such as cell proliferation and apoptosis. Our method postulates that even if the RCGs and a pathway may only have a few genes in common, they may be close to each other in the network. Although current compound pathway maps are incomplete, much relevant information is available in public databases of human molecular networks. While traditional pathway enrichment analysis methods like Fisher’s exact test identify pathways according to the number of shared genes, PACER prioritizes pathways based on their proximities to RCGs in molecular networks. Second, manually curated pathways may have arbitrary boundaries due to the need to capture knowledge at different levels of detail. Consequently, identifying drug-related pathways might be hindered by pathway boundaries. By leveraging the prior knowledge in molecular networks, PACER is more robust to the noise in pathway boundaries, thus improving the sensitivity of detecting compound-pathway associations.

We see many opportunities to improve upon the basic concept of PACER in future work. First, although the current PACER framework was developed in an unsupervised fashion, the scores assigned to each pathway for the given gene set can be used as the feature and plugged into off-the-shelf machine learning classifiers for compound-pathway association identification. Second, although this study focused on chemosensitivity response, the PACER method is broadly applicable to testing the association between two sets of genes according to their proximity in the network. Finally, although we use gene expression data as the molecular profile of each cell line, it might be interesting to test our method based on other molecular data such as somatic mutations and copy number alterations.

## Supporting information

S1 Text Supporting information. (DOCX)

S1 Table Genes that are significantly correlated with more than half of the compounds. (XLSX)

S2 Table Compounds that are significantly correlated with the most numbers of genes. (XLSX)

S3 Table Pathways significantly associated with the most numbers of compounds. (XLSX)

S4 Table Compounds significantly associated with the most numbers of pathways. (XLSX)

S5 Table Pathway compound associations that are predicted to be significant by PACER. (XLSX)
