# Supplementary material for: Identification of pathways associated with chemosensitivity through network embedding
Source: PLoS Comput Biol. 2019 Mar 20;15(3):e1006864. doi: 10.1371/journal.pcbi.1006864 (PMC6443184; doi:10.1371/journal.pcbi.1006864)
Supplement: S1 Text — (DOCX) [file pcbi.1006864.s001.docx]

###
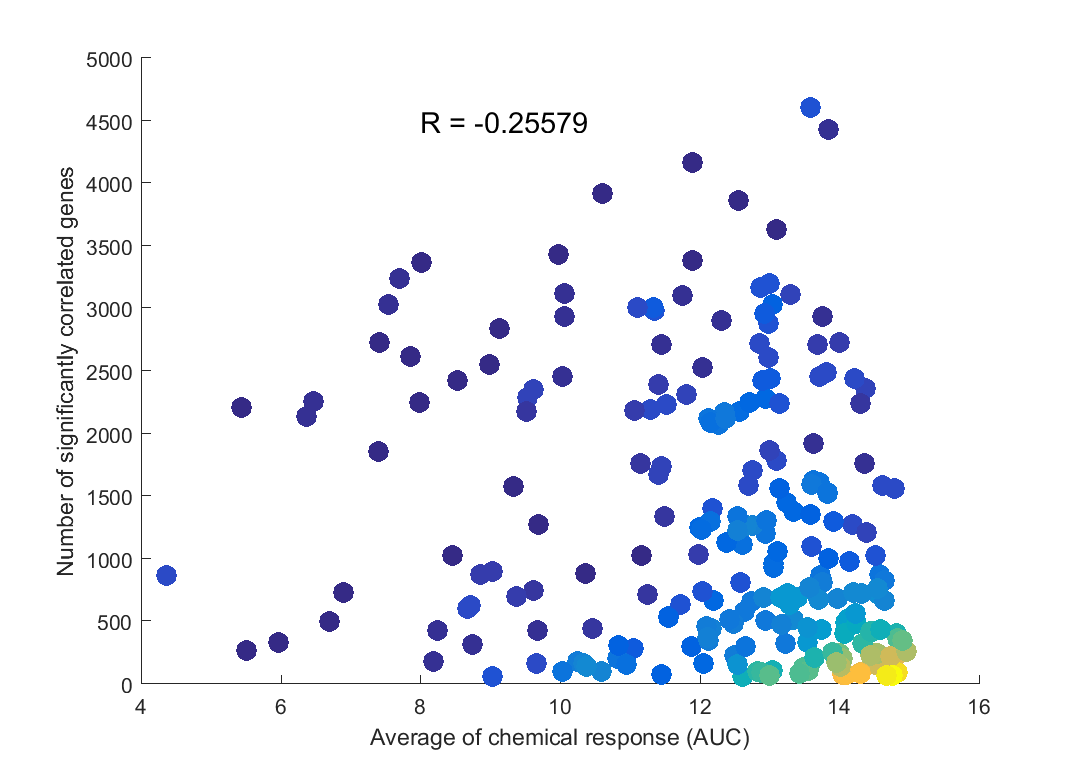


### **Suppl. Figure 1. Relationship between number of significantly correlated genes and average response to a compound. Spearman correlation coefficient: -0.26 (*p*-value:7.84e-05). Warmer color indicates a greater density.**


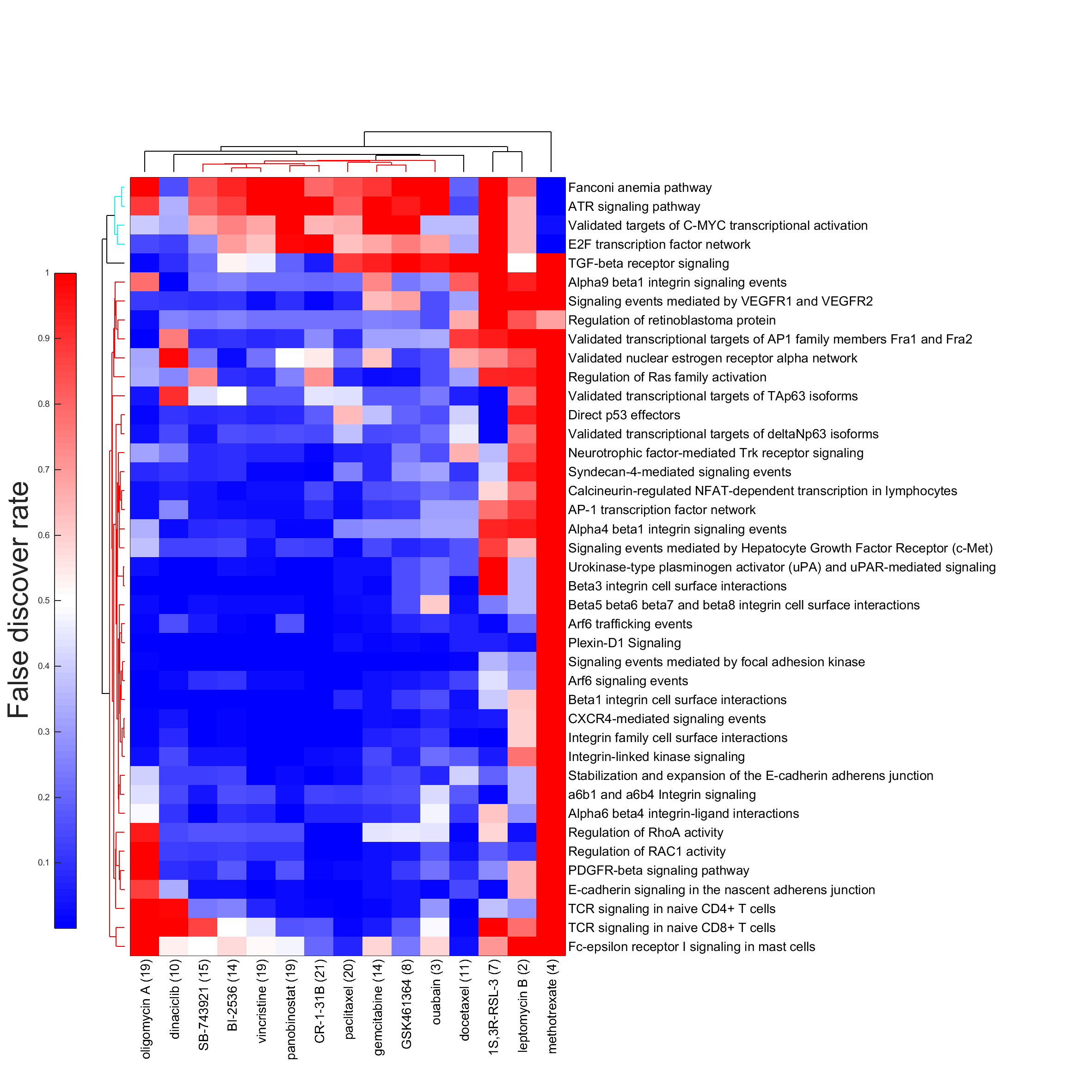


**Suppl. Figure 2. Heatmap of associations between compounds and pathways. Rows are pathways and columns are compounds.**


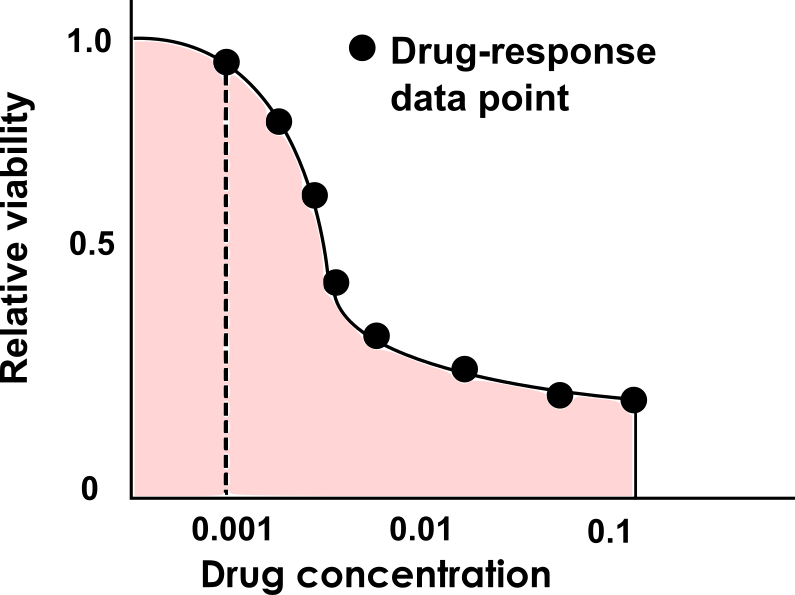


**Suppl. Figure 3. Illustration of the AUC value from Rees et al. drug response screen. The AUC value was measured over a 16-point concentration range and a curve was fitted by using a three-parameter sigmoid function. If the predicted EC50 was higher than the limit of drug exposure, another two-parameter sigmoid function was used to fit it so that the value of the lower asymptote is equal to the value of complete killing. AUC is calculated as the area under this curve. Here, each data point represents the relative viability *p* of a given cell line treated with a drug at concentration *q*, where *p* and *q* are shown on the *x*-axis and *y*-axis, respectively. These drug response data points form a drug response curve. AUC is thus the area under this drug response curve (pink area). A smaller AUC means the cell line has relatively smaller viability under the same concentration, which in turn indicates a more sensitive response to the drug. AUC is calculated from the cytotoxicity profile of each cell line treated with each drug. Therefore, the experimental data obtained from Rees et al. can be summarized in a 481 x 842 matrix of AUC values of each drug-cell line combination.**

###
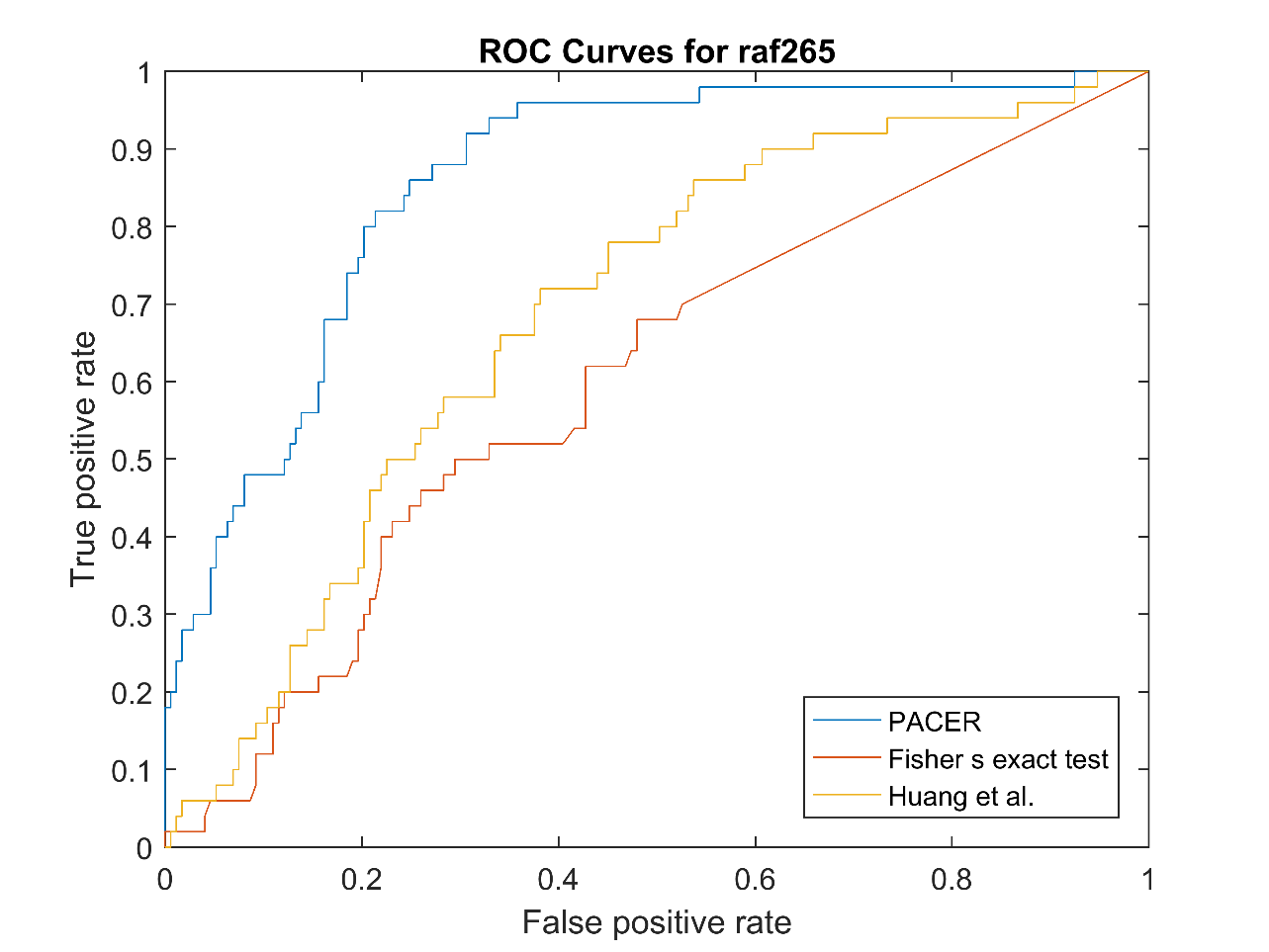


**Suppl. Figure 4. ROC curve of different method for raf265.**


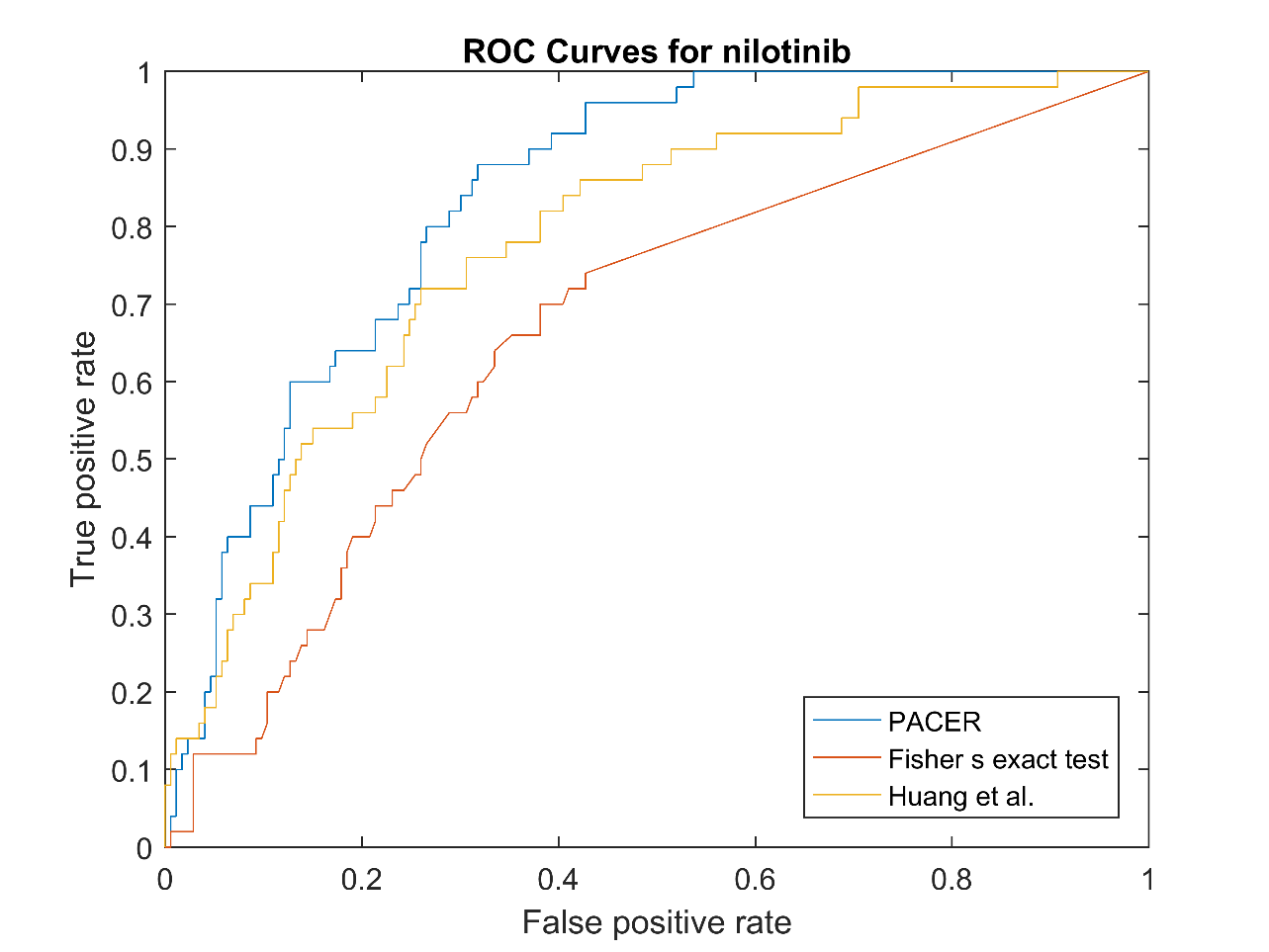


**Suppl. Figure 5. ROC curve of different method for nilotinib.**


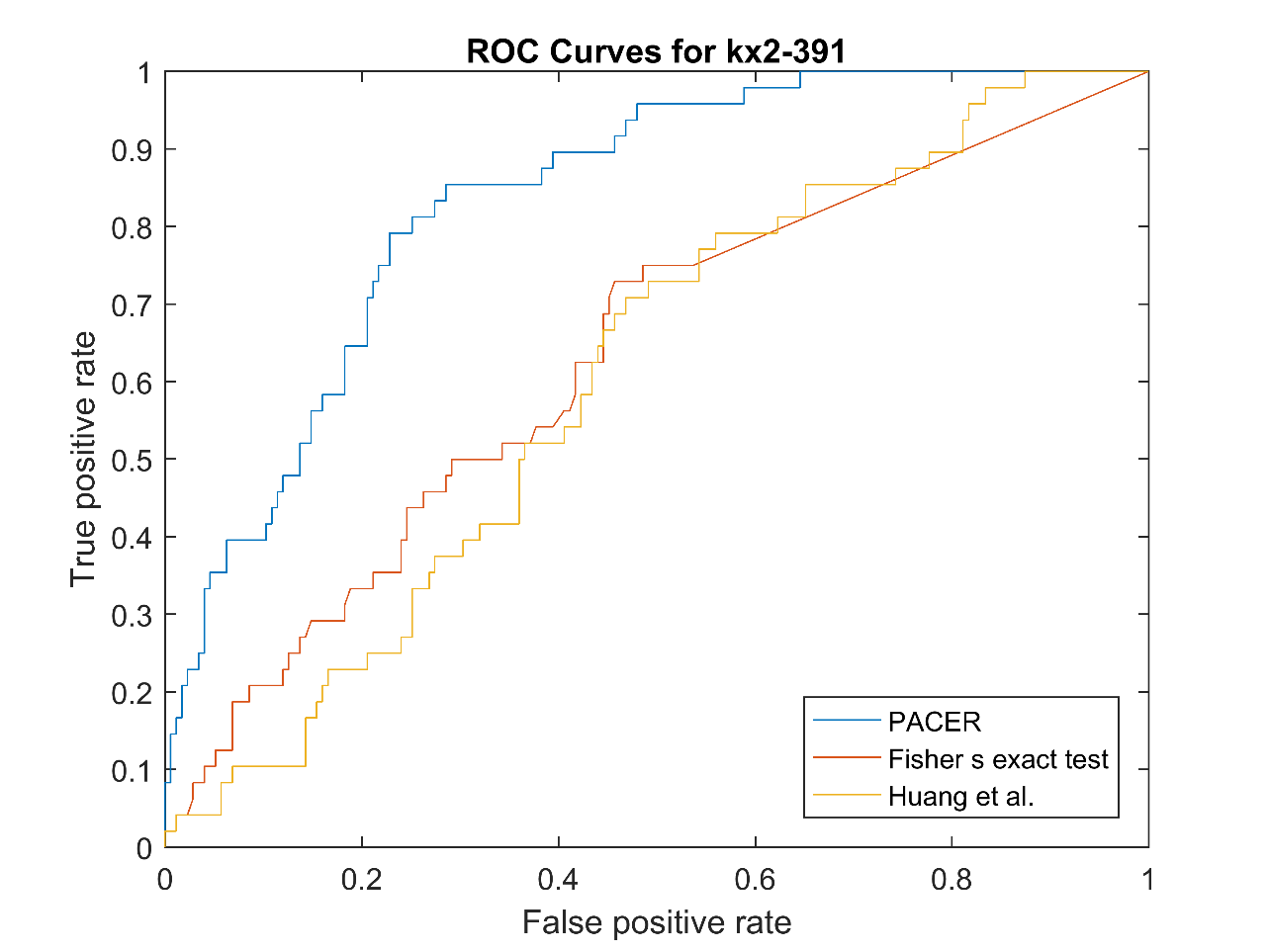


**Suppl. Figure 6. ROC curve of different method for kx2-391.**


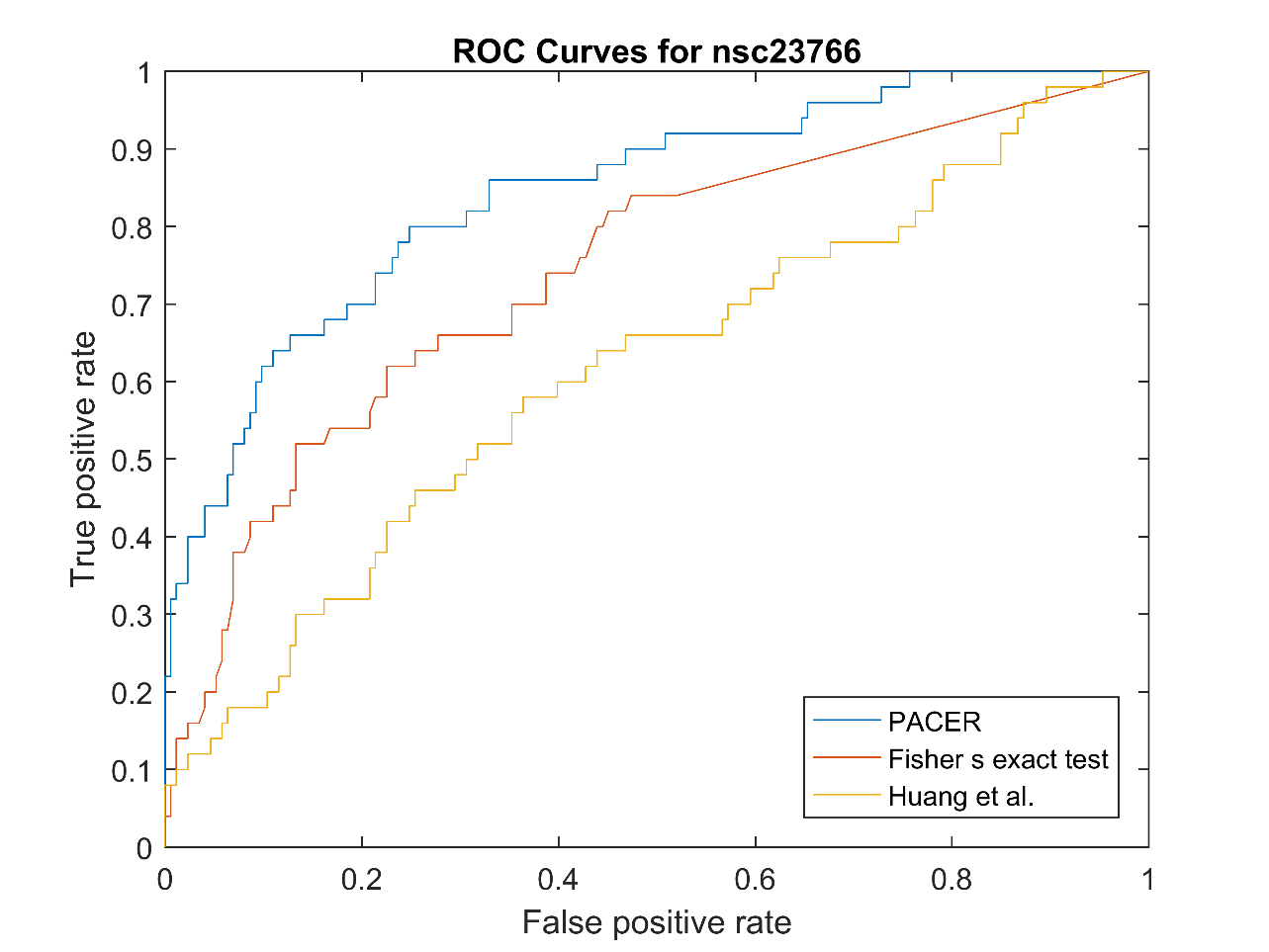


**Suppl. Figure 7. ROC curve of different method for nsc23766.**

**Suppl. Figure 8. Number of compounds with significant overlap (P < 0.05) between pathways from LINCS and pathways from PACER, from Huang et al. 2005 and from the baseline method (Fisher’s exact test) respectively, at different levels of stringency in pathway prediction. Only experimentally derived protein-protein interactions are used here. Stringency refers to the FDR control used by the baseline method in determining significant pathways. Both PACER and Huang et al. 2005 method were used to predict the same number of (highest scoring) pathways as the baseline method, for a fair comparison.**

**Suppl. Figure 9.** **Comparative evaluation of different methods for predicting compound-pathway associations. Only experimentally derived protein-protein interactions are used here. The ground truth used here is the pathways that contain any known target gene of the compound.**

**Suppl. Figure 10. Comparative evaluation of using different numbers of top response-correlated genes (‘k’ in ‘top k’) in PACER for predicting compound-pathway associations. Only experimentally derived protein-protein interactions are used here. The ground truth used here is the pathways that contain any known target gene of the compound.**

### ****

### **Suppl. Figure 11. Comparison of using different numbers of top response-correlated genes (‘k’ in ‘top k’) in finding compounds with significant overlap (P < 0.05) between pathways from LINCS and pathways from PACER at different levels of stringency in pathway prediction. Stringency refers to the FDR control used by the baseline method in determining significant pathways.**
